# IgA-Dominant Staphylococcus-Associated Glomerulonephritis: An Uncommon Complication of Intravenous Drug Use

**DOI:** 10.7759/cureus.52680

**Published:** 2024-01-21

**Authors:** Mason Stoltzfus, Pankhuri Mohan, Robert Mullin

**Affiliations:** 1 Internal Medicine, Penn State College of Medicine, Hershey, USA; 2 Internal Medicine and Pediatrics, Penn State Milton S. Hershey Medical Center, Hershey, USA

**Keywords:** iga-dominant infection-related glomerulonephritis, tricuspid valve endocarditis, acute hypoxemic respiratory failure, acute respiratory, pulmonary empyema, congestive hepatopathy, mrsa infective endocarditis, intravenous drug use (ivdu)

## Abstract

A 24-year-old female with a history of intravenous heroin use presented with two weeks of chills, myalgias, and cough and was found to be in acute hypoxemic respiratory failure. Subsequent workup revealed the presence of bilateral septic pulmonary emboli and tricuspid valve endocarditis. Several weeks into her hospitalization, she developed periorbital edema and laboratory testing revealed she had developed acute renal failure and nephrotic range proteinuria. A renal biopsy confirmed the diagnosis of IgA-dominant Staphylococcus-associated glomerulonephritis (IgA-SAGN). Early recognition of this newly recognized variant of glomerulonephritis is paramount, as improper treatment may lead to catastrophic consequences.

## Introduction

Infective endocarditis (IE) is the infection and subsequent inflammation of the endocardium. Risk factors for IE include dental procedures, heart valve dysfunction, congenital heart disease, the presence of a prosthetic valve, and intravenous drug use (IVDU) [1,2]. Right-sided IE is significantly less common than left-sided IE accounting for 5% to 10% of cases. IVDU is the leading cause of right-sided endocarditis. This is due to the fact that venous blood, which can be inoculated with bacteria during IVDU, passes through the right side of the heart prior to the left [3-5]. 

The incidence of IVDU has increased exponentially since the start of the opioid epidemic. The United Nations Office on Drugs and Crime reported that in 2022 as much as 0.28% of the global population aged 15 to 64 years used intravenous drugs [6]. From 2000 to 2013 the proportion of IE admissions related to IVDU increased from 6% to 12% [7]. Eighty-six percent of IE endocarditis cases linked to IVDU involve the right heart and 90 percent of those cases involve the tricuspid valve [5].

One of the uncommon sequelae of IVDU-related IE is post-infectious glomerulonephritis [2,8]. This type of glomerulonephritis is characterized by post-infectious glomerular damage due to the host's immune system triggering a cascade of responses driven by cytokines, effector cells, and the complement system. This inflammatory immune response causes migration of effector cells, particularly polymorphonuclear leukocytes, to the glomerulus where they trigger exudative damage to the endothelium. Furthermore, components of the complement system can bind to infectious antigens, forming immune complexes that deposit on, and damage, glomeruli. These mechanisms of glomerular injury can lead to a rapid decline in renal function and clinical status: prompt recognition and treatment of post-infectious glomerulonephritis is of paramount importance [8].

## Case presentation

A 24-year-old female with a three-month history of intravenous heroin use presented to the emergency department with two weeks of chills, myalgias, and a productive cough. On arrival, she was found to be in acute hypoxemic respiratory failure and had a white blood cell count of 14.9.

**Figure 1:**
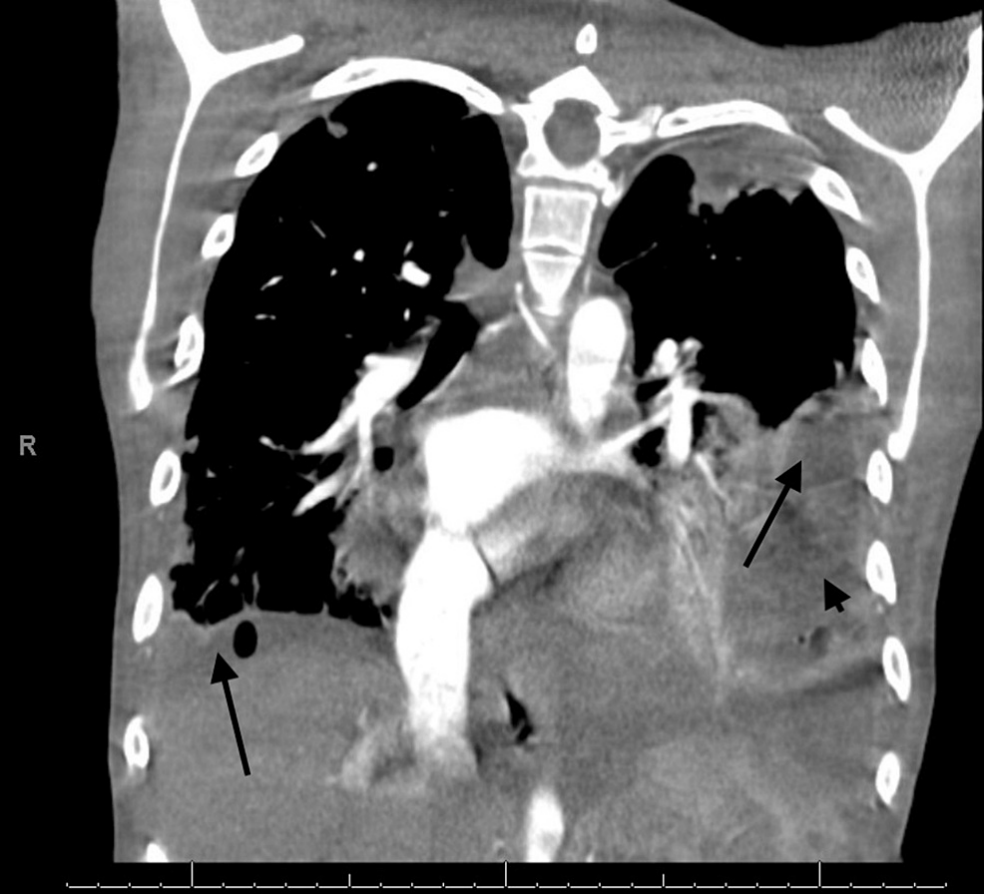
Coronal CT chest with contrast showing bilateral loculated pleural effusions (arrows) and left hydropneumothorax (arrowhead) secondary to septic pulmonary emboli.

She underwent a CT scan of the chest, which demonstrated multiple pulmonary emboli (Figure 1).

Blood cultures grew methicillin-resistant Staphylococcus aureus (MRSA). She was initially treated with vancomycin and meropenem but developed severe neutropenia, likely a rare reaction to these medications, prompting a transition to daptomycin and ceftriaxone for the remainder of treatment.

**Figure 2:**
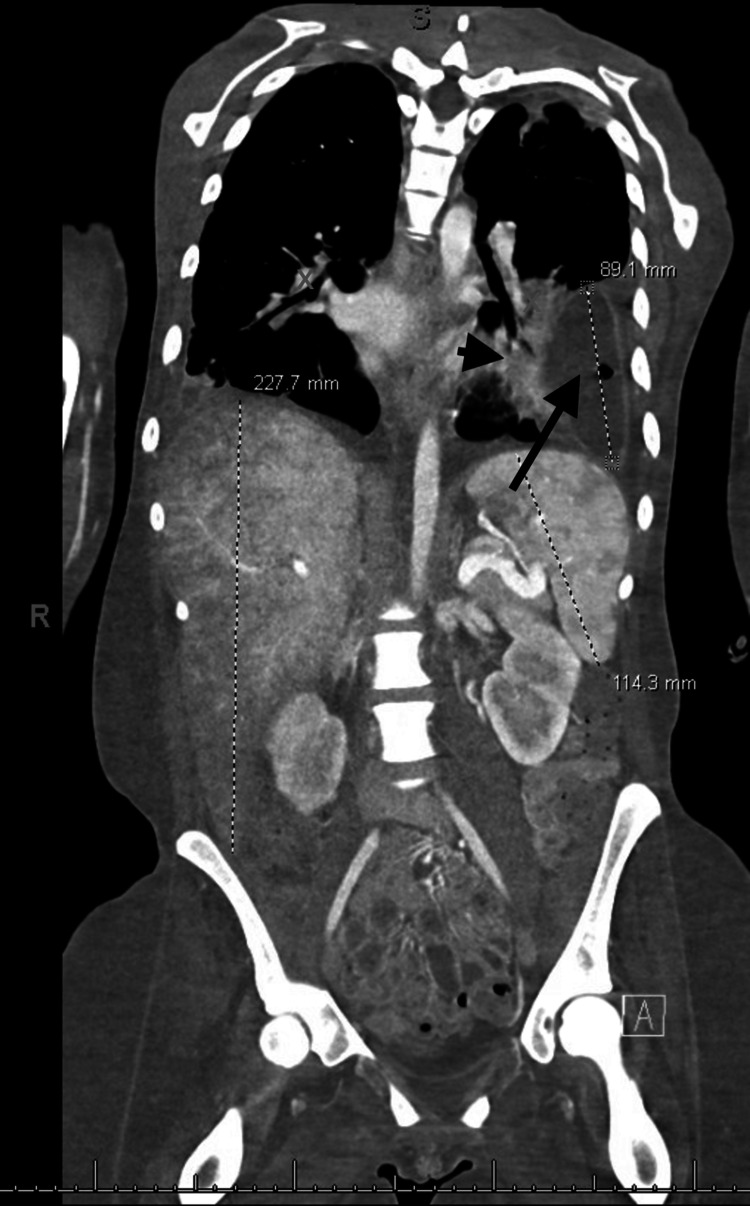
Coronal CT abdomen and pelvis with contrast showing septic emboli (arrowhead) and hydropneumothorax (arrow) consistent with empyema (measured 89.1 mm) in the left lower lung lobe.

Over the next few weeks, her course was complicated with worsening septic pulmonary emboli, loculated pleural effusions, and left hydropneumothorax (Figure 1 and Figure 2).

**Figure 3:**
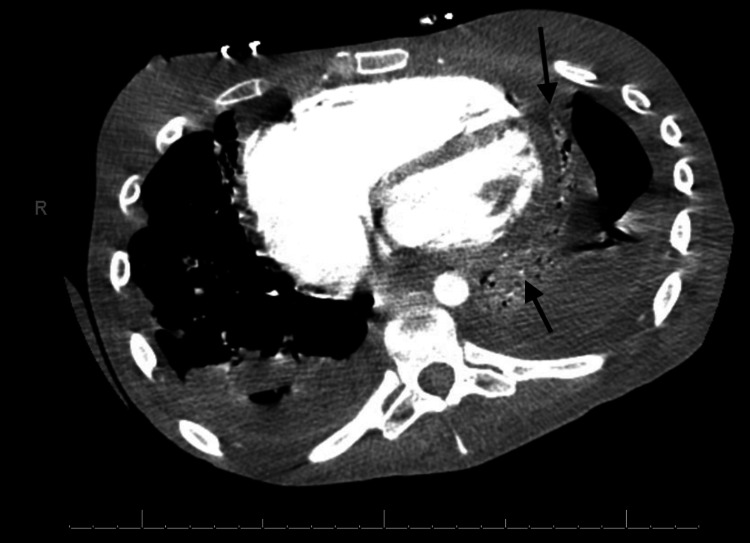
Axial CT chest with contrast showing pericardial effusion (arrows).

**Figure 4:**
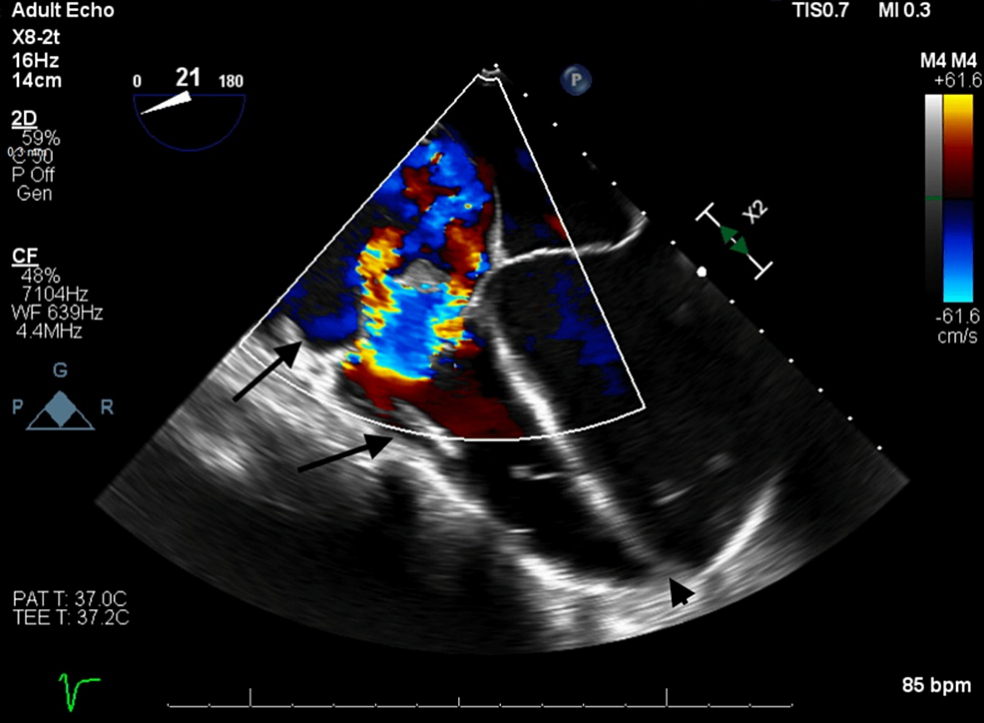
TTE showing torrential tricuspid regurgitation (colored), right atrial and ventricular dilation (arrows), and flattening of the intraventricular septum (arrowhead). TEE, transesophageal echocardiogram

This was followed by the development of large pericardial effusions (Figure 3) and acute systolic heart failure with transthoracic echocardiogram noting an ejection fraction of 10-15% (Figure 4).

**Figure 5:**
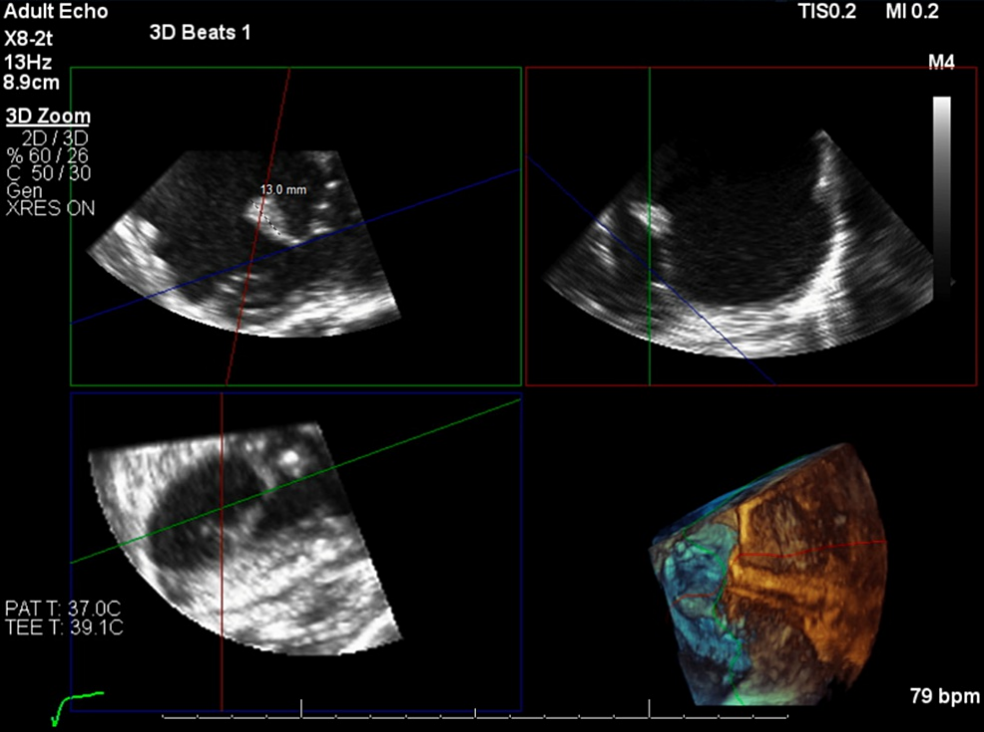
TEE showing a flail, anterior tricuspid valve leaflet with attached septic vegetation (13.0 mm measured structure). TEE, transesophageal echocardiogram

A transesophageal echocardiogram (TEE) also showed tricuspid valve endocarditis with a flail, anterior tricuspid leaflet with a septic vegetation (Figure 5).

This contributed to the development of congestive hepatosplenomegaly (Figure 3). She was started on guideline-directed medical therapy with metoprolol succinate, lisinopril, and furosemide.

Several weeks into the hospitalization, she developed anasarca and significant periorbital edema. Her BUN/creatinine was 20/0.82, compared to 11/0.60 at admission. Her GFR was also now 102 mL/min, compared to 104 mL/min at admission. This prompted a urinalysis, which was significant for 10-15 red blood cells and 40-50 mg/dL protein. She had a urine protein creatinine ratio (UPCR) of 4.5 prompting a workup for nephrotic range proteinuria. Notably, her workup was revealing for extensive mesangial proliferation with IgA and C3 deposition in her renal biopsy, raising suspicion of IgA nephropathy or infection-related glomerulonephritis. In addition, she had evidence of moderate acute tubular injury thought to be a sequelae of glomerulonephritis or a result of hypotension or diuresis. The rest of the workup for her renal injury and nephrotic-range proteinuria was negative, including negative hepatitis panel, HIV, ANA, ANCA, anti-dsDNA, and normal C3 and C4. Her clinical presentation was thought to be consistent with IgA-dominant staphylococcal infection-associated glomerulonephritis. She remained on broad-spectrum antibiotic therapy and was continued on lisinopril in an effort to reduce intraglomerular pressure and subsequently try to improve her nephrotic range proteinuria. 

Following three months of aggressive antibiotic therapy, she was transitioned to oral doxycycline for long-term suppressive antimicrobial therapy. She was subsequently discharged to inpatient rehab to address her severe deconditioning.

The patient was unable to maintain sobriety and returned to using intravenous drugs shortly after discharge from inpatient rehab. Approximately six months later, she presented to the hospital in cardiogenic shock and was found to have methicillin-sensitive Staphylococcus aureus bacteremia as well as recurrent tricuspid valve endocarditis and pulmonary valve endocarditis. She was ultimately transferred to a different tertiary care center where she underwent emergent bioprosthetic tricuspid and pulmonic valve replacements.

## Discussion

The microbe causing this patient’s IE was methicillin-resistant Staphylococcus aureus. Historically a rare culprit of IE, it has increased in prevalence due to the rising popularity of IVDU in the late 20th century [9]. IE can be difficult to diagnose, but this patient’s clinical history of intravenous heroin led her team to the diagnosis. In addition to clinical history, a TEE is the gold-standard diagnostic tool to visualize the heart valves and associated vegetation [3,10].

This patient’s intravenous heroin use inoculated her with MRSA, leading to IE and the development of valvular vegetation. These heart valve vegetations then pose the risk of rupturing and embolizing the lungs as demonstrated by this case. The combination of intensive antibiotic therapy and tricuspid valve replacement is the only definitive treatment for this condition.

Acute post-infectious glomerulonephritis (APIGN) is a rare phenomenon that can occur in patients with IE. Typically, APIGN is associated with post-streptococcal glomerulonephritis (PSGN), which most commonly affects children after a streptococci pharyngeal infection. More recently, it has been discovered that APIGN can be associated with a variety of bacterial, viral, and protozoal infections. The incidence of APIGN has declined precipitously in developed countries in recent decades [11]. Nasr et al. study of 86 adults with APIGN found that IE was associated with 11.6% of cases, and IVDU was associated with 1.2% of cases of APIGN [2].

IgA-dominant Staphylococcus-associated glomerulonephritis (IgA-SAGN) is a subtype of APIGN. IgA-SAGN is a rapidly progressive glomerulonephritis that most commonly affects older adults (mean age: 59) with male predominance [8,12]. Those with IgA-SAGN often develop a vasculitis-like skin rash similar to the rash seen in IgA vasculitis, otherwise known as Henoch-Schönlein purpura (HSP) [12]. Labs are often notable for the rapid development of an acute kidney injury with various degrees of proteinuria and hematuria as well as evidence of ongoing infection [13]. In comparison to other types of APIGN, those with IgA-SAGN typically have normal complement levels [12]. Although the pathogenesis is not well understood, IgA-SAGN is thought to involve glomerular deposition of circulating immune complexes. which remain in the bloodstream for a prolonged period, increasing their chance of forming complexes and depositing in the glomerulus. There is also a postulation that MRSA antigens may act as "super antigens," which cause increased T-cell activation and subsequent B-cell production of IgA, IgG, and IgM antibodies. IgA antibodies have been found to form immune complexes with MRSA antigens more readily, ultimately leading to glomerular IgA immune complex deposition [13,14]. Histologically, IgA-SAGN appears similar to primary IgA nephropathy with mesangial deposition of IgA immune complex [11,12].

Although IgA-SAGN may present similarly to primary IgAN and HSP, it is imperative to correctly differentiate between them as they require different treatment approaches. Due to the presence of underlying infection, the treatment for IgA-SAGN involves antibiotic therapy and supportive care with a low-sodium diet and diuretics as needed [14,15]. On the other hand, HSP and primary IgAN are treated with supportive care, corticosteroids, and/or immunomodulators depending on their renal function and severity of proteinuria. Immunosuppressive therapy in the setting of IgA-SAGN is not well studied, and there is no clear evidence of benefit [14,15]. In addition, in patients with active infection, as is often the case in patients with IgA-SAGN, giving high-dose glucocorticoids may potentially lead to clinical deterioration or even death.

Our approach to this case was weakened by the fact that IgA-SAGN was not part of our differential diagnosis until the patient experienced significant clinical deterioration due to renal damage. This was likely due to our patient's myriad of comorbidities. In patients with MRSA septicemia, it is imperative to suspect possible IgA-SAGN to effectively differentiate it from other disorders like HSP, treat it, and prevent deterioration.

## Conclusions

This case report highlights the clinical course of a young patient who developed IE from IVDU and is useful for predicting clinical courses and constructing differential diagnoses for patients with similar presentations and risk factors. Tricuspid valve endocarditis is a well-documented disease that can occur secondary to IVDU. This is particularly important because IE can predispose to septic valvular vegetation deposition, heart valve failure, and heart failure with a reduced ejection fraction. Furthermore, despite these significant cardiac and pulmonary complications, it is crucial to also monitor for the presence of infection-associated glomerulonephritis as this can contribute to significant morbidity and mortality if left unrecognized or untreated. For patients with MRSA septicemia, it is imperative to maintain an appropriate level of suspicion for IgA-SAGN. IgA-SAGN can present with a vasculitis-like skin rash and acute kidney injury with nephrotic syndrome and hematuria. Making the correct diagnosis is key, as this disease mimics others, such as HSP, which require different treatment plans that may exacerbate patients' conditions if mistreated.
